# Microscope calibration protocol for single-molecule microscopy

**DOI:** 10.1364/OE.408361

**Published:** 2020-12-22

**Authors:** Sungyong You, Jerry Chao, Edward A. K. Cohen, E. Sally Ward, Raimund J. Ober

**Affiliations:** 1 Department of Biomedical Engineering, Texas A&M University, College Station, TX 77843, USA; 2 Department of Molecular and Cellular Medicine, Texas A&M University Health Science Center, College Station, TX 77843, USA; 3 Astero Technologies LLC, College Station, TX 77845, USA; 4 Department of Mathematics, Imperial College London, London SW7 2AZ, UK; 5 Centre for Cancer Immunology, Faculty of Medicine, University of Southampton, Southampton SO16 6YD, UK

## Abstract

Single-molecule microscopy allows for the investigation of the dynamics of individual molecules and the visualization of subcellular structures at high spatial resolution. For single-molecule imaging experiments, and particularly those that entail the acquisition of multicolor data, calibration of the microscope and its optical components therefore needs to be carried out at a high level of accuracy. We propose here a method for calibrating a microscope at the nanometer scale, in the sense of determining optical aberrations as revealed by point source localization errors on the order of nanometers. The method is based on the imaging of a standard sample to detect and evaluate the amount of geometric aberration introduced in the optical light path. To provide support for multicolor imaging, it also includes procedures for evaluating the geometric aberration caused by a dichroic filter and the axial chromatic aberration introduced by an objective lens.

## 1. Introduction

Single-molecule wide-field microscopy experiments have been widely used for a broad range of investigations in cell-biological studies [1]. Two of the most important types of such single-molecule experiments are single-molecule tracking and localization-based super-resolution experiments. Single-molecule tracking experiments hold the promise to reveal fundamental insights into dynamic molecular processes in live cells that remain difficult to uncover using classical microscopy approaches [2–5]. Localization-based super-resolution microscopy can yield quantitative information on the spatial distribution of a molecule of interest and the spatial characteristics of cellular structures smaller than the diffraction limit [6–9]. Both types of experiments importantly depend on the estimation of the position of single molecules with a low level of uncertainty (i.e., a small variance or standard deviation) [10].

Localization of single molecules with statistical uncertainties of tens of nanometers is routinely achievable in single-molecule microscopy [11,12], and sub-nanometer uncertainty is even possible when using fluorophores that emit large numbers of photons [13,14]. Being able to achieve sub-nanometer uncertainty is critical for our current purposes, as we seek to assess the performance of microscopes and optical components by determining differences in the positional estimates of imaged point sources that will typically be on the order of nanometers.

The complexity of advanced microscopy setups, optical imperfections, and possible misalignments of mirrors or lenses can all contribute to geometric aberrations in the image produced by a microscope. Errors due to such non-ideal conditions can lead to erroneous answers to biological questions, even when they are on the nanometer scale. For example, when imaging the dynamics of a single molecule that interacts with an endosome, the position of a single molecule may be determined to be inside rather than outside of the endosome, or vice versa, when the localization error is on the order of only nanometers. Errors of this nature can obviously lead to vastly different interpretations of the biological phenomenon. Therefore, to ensure that data of reliably good quality is produced, the performance of the imaging system must be routinely assessed prior to performing single-molecule imaging experiments. This assessment requires defined samples that can be used as standards and calibration methods that are suited for single-molecule localization microscopy. The calibration methods should be system-independent and yield reproducible results, such that the same standards can be used to calibrate different microscopes.

Several calibration standards have been introduced over the years to characterize the performance of an imaging system, including ones that utilize fluorescent beads [15,16] and DNA origami [17,18]. In approaches that make use of beads, the samples are typically prepared by mounting randomly distributed small fluorescent beads (0.1 µm in diameter) onto the cover glass. For example, 0.1 µm TetraSpeck fluorescent beads (Thermo Fisher Scientific) are one of the common standards for fluorescence microscopy and have four well-separated pairs of excitation and emission peaks located at 350/440 nm (blue), 505/515 nm (green), 575/585 nm (orange) and 655/685 nm (dark red). Their ability to produce fluorescence of different colors makes them a particularly good calibration standard for multicolor applications. However, fluorescent beads can form aggregates, leading to quenching and altered spectroscopic properties. An approach using beads also has limited sampling uniformity in terms of the size and distribution of the beads. Unlike with fluorescent beads, with DNA origami the distribution of the fluorophores can be controlled on the sub-micrometer level. However, the emitter intensity and stability are limited and, furthermore, it may be challenging to create a DNA origami sample occupying a field of view that is large enough to capture at least an entire biological cell of interest (i.e., at least tens of micrometers per lateral dimension).

To overcome the various limitations of approaches that use beads or DNA origami, in recent years techniques have been developed to create slides imprinted with defined patterns for system calibration. For example, Argolight uses a laser to induce into glass substrates fluorescent materials that are stable and have a broadband emission spectrum. An example of an Argolight slide contains different fluorescent patterns in two and three dimensions, the elementary structure of which is an empty cylinder with a diameter of about 0.7 µm. A second technique is the lithography system used by Miraloma Tech to produce calibration slides containing defined patterns. A particularly useful pattern consists of an array of regularly spaced sub-wavelength-sized apertures, allowing for uniform sampling of the imaging field. These apertures are empty and imaged using trans-illumination. Hence the imaging of these slides is not affected by fluorophore photobleaching and can produce extremely high signal-to-noise ratio images even with short exposure times.

Recent studies have used arrays of regularly spaced points (i.e., small apertures or fluorescent features) or other patterns or features to evaluate the performance of fluorescence microscopes in terms of lateral resolution, field distortion, chromatic aberration, etc. In [19], arrays of small apertures, fabricated using electron-beam lithography and filled with fluorescent dyes, are used to measure field-dependent variations in the three-dimensional (3D) point spread function of a microscope, which have direct implications on the accuracy of the 3D localization of single molecules. In [20], laser-written fluorescent patterns are utilized for evaluating microscope properties such as illumination uniformity and chromatic alignment. In [21], arrays of circular apertures, fabricated using electron-beam lithography, are used to determine the field curvature, assess chromatic aberration, and evaluate other aspects of a microscope.

We will similarly make use of an array of sub-wavelength-sized apertures in this study. The basis of our microscope calibration method is the evaluation of geometric aberrations by way of determining the difference between the true and the measured positions of the apertures. An important issue we take into account is that there can be manufacturing errors associated with the positions and sizes of the apertures. Our method determines the true aperture positions as a set of reference positions, as relying on the nominal aperture positions would make it difficult to determine whether the geometric aberrations observed in the acquired images are due to defects in the optical system or manufacturing errors in the calibration sample.

Recently, simultaneous multicolor single-molecule tracking has been widely used for the observation of the fast dynamics of individual molecules in living cells [3,22,23]. For these experiments, the imaging system requires the use of dichroic filters to separate signals of different colors. However, previous studies have not evaluated how much the image quality is affected by geometric aberrations due to the surfaces of the dichroic filters being insufficiently flat. In addition, few methods have been introduced to evaluate the objective lens in terms of the chromatic aberration that it introduces.

Therefore, the calibration method that we present here is optimized for multicolor single-molecule microscopy. This method couples the use a lithographically fabricated aperture array with novel analysis algorithms to evaluate the performance of the microscope and its optical components with high accuracy.

The proposed method comprises two major components. The first component concerns the assessment of the performance of a microscope through determining the level of geometric aberration in its optical path. As the extent of the aberration is reflected in the deviation of the imaged positions of microscopic apertures in the calibration sample from their true positions, the key here, as mentioned above, is to establish a set of accurately determined reference aperture positions that serve as a benchmark for comparison in subsequent calibrations of a microscope using the same sample. The approach therefore entails procedures for the accurate estimation of imaged aperture positions and the determination of the similarity between a given set of aperture position estimates and the reference aperture positions.

The second component of the proposed calibration method comprises approaches for evaluating two critical microscope components, namely the objective lens and the dichroic filter. The quality of the objective lens is evaluated based on how well it corrects for chromatic aberration along the optical axis. This assessment is important because there is no guarantee that even apochromat objectives will be able to fully correct for the aberration over a wide spectral range. The performance of the dichroic filter is evaluated by investigating the deviation between corresponding aperture positions estimated from images simultaneously acquired of the filter’s transmitted and reflected light paths. This type of evaluation is of particular importance because it is often the case that suboptimal properties of a dichroic filter lead to distortions in the light that it reflects.

The proposed calibration method is supported by a core set of analysis tools for carrying out a number of important tasks. These tasks comprise the determination of the in-focus position of a 3D data set, the registration of images in two dimensions, the estimation of the position of an aperture, the assessment of the horizontality of the microscope stage and calibration sample, and the quantitative comparison of two data sets.

To demonstrate the evaluation of the performance of a microscope, we apply our calibration method to both a microscope of poor quality (one with a damaged objective thread adapter) and a microscope of good quality. Our results show that our method is capable of distinguishing between the two microscopes in terms of the level of geometric aberration.

In addition, we apply our method to an apochromat objective lens to evaluate its axial chromatic aberration over a wide spectral range. We also demonstrate the ability of our method to distinguish between dichroic filters that differ in terms of their flatness.

The proposed method enables meaningful and repeatable calibration data acquisitions that are reproducible across different microscopes. Also, our approach provides useful insights in the selection of an appropriate microscope and suitable optical elements for a given application. It allows researchers, ranging from novice microscope users to expert microscopists, to evaluate their microscopes and optical elements with high accuracy.

## 2. Methods

We describe in this section the type of sample, instrumentation, and data that are required by our calibration method, as well as the specific implementations that are used in Section 3 to demonstrate the method. We also detail here the protocols that comprise our method, including a core set of analysis tools on which the protocols rely. Although it is not a part of the calibration method, we additionally present here a procedure to simulate data sets for the investigation of a measure that we introduce for the comparison of two data sets.

### 2.1 Calibration sample

A NanoGrid slide (Miraloma Tech, LLC) is used as a calibration standard for the microscope performance evaluation. The slide consists of a 20 by 20 array of sub-wavelength-sized apertures with a regular spacing of 4 µm between adjacent apertures. The apertures, which we will also refer to as holes, are approximately 200 nm in diameter. The numbers for the spacing and diameter are taken from specifications provided by the manufacturer. Of the 20 by 20 array of holes, only the center 10 by 10 holes are used for the proposed analyses (except for the determination of the in-focus image of a z-stack (Section 2.4), which is carried out using the whole image). The field of view corresponding to the center 10 by 10 holes is large enough to capture a single cell of interest in its entirety (which is typically all that is needed in single-molecule imaging), and yet small enough to help speed up the rate of image acquisition.

### 2.2 Microscope setups

We use the following three microscope configurations for the current study. The first microscope configuration (“microscope 1”) consists of a standard inverted microscope (Axio Observer A1, Carl Zeiss) and an EMCCD camera (iXon Ultra, Andor) operated in CCD readout mode. The camera exposure time is set to 0.1 s. The plan-apochromatic objective lens (Cat. No. 420782-9900-000, Carl Zeiss) has a 63× magnification and a numerical aperture of 1.4 and is used with an immersion medium with an index of refraction of 1.51. A piezo objective positioner (P-721, Physik Instrumente) is used to translate the objective lens in the z direction. This device has a positioning repeatability (i.e., the precision for attaining a target position under identical conditions) of ±5 nm. A microscope objective thread adapter (Cat. No. 000000-1095-168, Carl Zeiss) is installed between the objective lens and the piezo objective positioner. This adapter allows microscope objectives with RMS (Royal Microscopical Society) objective mounts to be used on piezo objective positioners with M27 (metric 27-millimeter) threads. To provide an example of a microscope with geometric aberrations, the adapter that is installed is replaced with one whose thread is damaged. The damaged thread causes the objective lens to deviate from its alignment with the optical axis, resulting in aberrations in the image formed.

The second microscope configuration (“microscope 2”) consists of a standard inverted microscope (Axio Observer Z1, Carl Zeiss) and a CCD camera (ORCA-ER, Hamamatsu Corporation). The camera exposure time is set to 0.2 s. The plan-apochromatic objective lens (Cat. No. 420782-9900-000, Carl Zeiss) has the same magnification and numerical aperture as the objective lens for the first setup and is used with the same immersion medium. A motorized focus drive included in the microscope stand is used to translate the objective lens along the optical axis. For chromatic aberration analysis, microscope 2 is equipped with filter sets for FITC (Filter set 38, Carl Zeiss), Cy3 (Filter set 43, Carl Zeiss), and Cy5 (Filter set 50, Carl Zeiss).

The third microscope configuration (“microscope 3”) is assembled by adding an emission image splitter (Cat. No. 1058640000, Carl Zeiss) to microscope 1. Two identical EMCCD cameras (iXon Ultra, Andor), both operated in CCD readout mode, are connected to the output ports of the image splitter unit. The exposure time is set to 0.1 s for each camera. For the evaluation of dichroic filters, either a standard flatness dichroic filter (FF625-SDi01, Semrock Inc) or an improved flatness dichroic filter (FF560-FDi01, Semrock Inc) is mounted in a filter cube and placed in the image splitter unit.

Briefly, after adding the image splitter to the microscope and attaching the two cameras, the following procedure is performed to align the in-focus positions of the two cameras. A filter cube with a 50:50 beam splitter is inserted into the image splitter, and a NanoGrid slide is positioned so that its image is placed in the center of the field of view of the camera in the transmitted light path. If necessary, the xy position of the camera in the reflected light path is then adjusted (using position adjustment knobs that are part of the image splitter) so that the image of the slide is also centered in its field of view. To check whether the two cameras share a focal plane, z-stacks of the NanoGrid sample are simultaneously acquired using the cameras, and the procedure of Section 2.4 is used to analyze each z-stack to determine the in-focus position of its respective camera. If the in-focus positions of the two cameras do not match well, then the z position of the camera in the reflected light path is adjusted as needed, again using the adjustment knobs. The z-stack acquisition and analysis are repeated until the two cameras are found to share a focal plane.

The image splitter used in microscope 3 is a discontinued product. A similar product that could be used is the Double Adapter Duolink (Cat. No. 426143-9000-000, Carl Zeiss) [24].

In all three microscope setups, the NanoGrid calibration slide is trans-illuminated with a light-emitting diode (LED) (M810L3-C4, Thorlabs Inc). Also, the mechanical stage (Mechanical stage 130 × 85 R/L with short coaxial drive, Carl Zeiss) is equipped with a mounting frame (Universal Mounting Frame K, Carl Zeiss) that can hold a petri dish (max. diameter 68 mm) or a sample slide (max. length 120 mm). The mounting frame can be detached from the stage and rotated 180 degrees.

### 2.3 Acquisition of calibration data

In this paper, three types of calibration data sets are utilized. The first type (“calibration data set 1”) consists of a z-stack of the NanoGrid slide, the second type (“calibration data set 2”) comprises multicolor z-stacks of the slide, and the third type (“calibration data set 3”) consists of 200 in-focus images of the slide.

Calibration data set 1 is acquired as follows. The aperture array on the calibration slide is placed at the center of the imaging field. A z-stack is then acquired by moving the objective lens along the optical axis to obtain a series of images centered about the visually determined in-focus position. More specifically, the z-stack is obtained by moving the objective lens with a piezo nanopositioner or a motorized focus drive in 50-nm steps and acquiring an image at each position of the objective lens. Calibration data set 2 is acquired using the same procedure, except an image of each of the color channels is taken per z-step using a motorized filter cube turret.

Calibration data set 3 is acquired as follows. A z-stack is first acquired using the procedure for the acquisition of calibration data set 1. The in-focus frame number of the z-stack is determined as described in Section 2.4 and the in-focus position of the objective lens is obtained as the product of this frame number and the step size used to acquire the z-stack. The objective lens is then moved to the in-focus position and 200 images are acquired sequentially.

### 2.4 Determination of the in-focus position of a z-stack

The Brenner gradient method [25,26] is used to determine which image amongst a z-stack of images (i.e., from calibration data set 1 or 2) is closest to being in-focus. The method is a computationally efficient edge detector that measures the intensity difference between a pixel and a neighboring pixel. When plotted as a function of the frame number in a z-stack, the Brenner gradient exhibits a sharp peak at the in-focus position and drops rapidly away from the in-focus position. For each image in a z-stack, the Brenner gradient is computed as

$$F_{Brenner}=\sum_{i=1}^{M-m}\sum_{j=1}^{N}{\left[I(i,j)-I(i+m,j)\right]}^{2},$$

where I(i,j) is the intensity of pixel (i,j), M and *N* are the height and width of the image in pixels, and m=2. Each Brenner gradient value $F_{Brenner}$ is normalized using the formula

$$F_{norm}=(F_{Brenner}-F_{\min})\times \frac{1}{F_{\max}-F_{\min}},$$

where $F_{\min}$ and $F_{\max}$ are the smallest and largest of the gradients calculated for all images in the stack. The Brenner gradient for each frame in the z-stack is therefore converted to a value between 0 and 1. To find the in-focus frame number, the normalized gradient values $F_{norm}$ are plotted as a function of the frame number in the z-stack. The plot is smoothed by interpolating the data points around the maximum of the plot with a fourth-order polynomial. The frame number closest to the maximum of the interpolating polynomial is taken to be the in-focus frame number of the z-stack.

### 2.5 2D image registration

The image registration employed by our calibration method is concerned with affine transformations such as translation, scaling, and rotation. We assume that there exists, between two different images, an affine transformation

$$T:{\mathbb{R}}^{2}\to {\mathbb{R}}^{2},x\mapsto Ax+d,$$

where $A\in {\mathbb{R}}^{2\times 2}$ is a square invertible matrix and $d\in {\mathbb{R}}^{2\times 1}$ is a translation vector. We assume that *A* can be written as

A=cR,

where $R\in {\mathbb{R}}^{2\times 2}$ is a rotation matrix and c>0 is a scale factor. Here *R* is rigid (i.e., $R^{T}R=I$) and there is no reflection (i.e., det(R)=1). The translation vector *d* and rotation matrix *R* are explicitly given by

$$d=\left[\begin{array}{c} d_{x}\;\\ d_{y} \end{array}\right],\;R=\left[\begin{array}{cc} \cos\phi & -\sin\phi \\ \sin\phi & \cos\phi \end{array}\right],$$

where $d_{x}$ and $d_{y}$ are the translations in the x and y directions and −π<ϕ≤π is the angle of the rotation. To determine the transformation from a source image to a target image, our task is to estimate the parameter vector $\theta =(\phi ,d_{x},d_{y},c)$. Generalized least squares is the correct and optimal statistical procedure for estimating θ [27], as it is unbiased and has minimum variance. For rigid transformations this gives the estimate $\hat{\theta }$ of θ to be

$$\hat{\theta }=(\hat{\phi },{\hat{d}}_{x},{\hat{d}}_{y},\hat{c}\;)=\underset{\theta }{\mathrm{arg min}}\;tr(WW^{T}),$$

where $W={\left(1+c\right)}^{-1/2}(t-d1_{n}^{T}-cR(\phi )\;s)$, *n* is the number of source points as well as the number of target points used to determine the transformation, $1_{n}$ is an n×1 vector of ones, $s=\left[\begin{array}{ccc} x_{1,src} & \cdots & x_{n,src}\\ y_{1,src} & \cdots & y_{n,src} \end{array}\right]\in {\mathbb{R}}^{2\times n}$ is a matrix of the estimated 2D positions $\left(x_{1,src},\;y_{1,src}),(x_{2,src},y_{2,src}),\ldots ,(x_{n,src},\;y_{n,src}\right)$ of the *n* points from the source image, and $t=\left[\begin{array}{ccc} x_{1,trgt} & \cdots & x_{n,trgt}\\ y_{1,trgt} & \cdots & y_{n,trgt} \end{array}\right]\in {\mathbb{R}}^{2\times n}$ is a matrix of the estimated 2D positions $\left(x_{1,trgt},y_{1,trgt}),(x_{2,trgt},y_{2,trgt}\right)$, $\ldots ,(x_{n,trgt},y_{n,trgt})$ of the *n* points from the target image. In the context of our calibration method, the source and target points are the NanoGrid apertures used for analysis.

### 2.6 Estimation of hole positions

To estimate the axial position of each hole in calibration data set 1 or 2, the in-focus image of the z-stack is first determined as described in Section 2.4. From the in-focus image, the imaged holes are detected by a wavelet-based segmentation algorithm [28]. For each detected hole, the region of interest (ROI) is defined as a sub-region centered on the brightest segmented pixel. The ROIs defined in the in-focus image are applied to the other images in the z-stack. To avoid overlap between adjacent ROIs, the ROI size is designed according to the camera pixel size and the distance between two adjacent holes. At the same time, one must also make sure that the ROI size is sufficiently large, such that the images of the NanoGrid holes are well within their confines in every frame of the z-stack. To calculate the axial position of each hole, the same analysis as described in Section 2.4 is applied to the ROI corresponding to each hole. However, the z position of the maximum of the interpolating polynomial is taken to be the axial position of the hole in this analysis. Assuming the number of the first frame of the z-stack is set to 1, the z position of a hole is estimated as $z_{pos}=(f_{\max}\;-1)\times z_{step}$, where $f_{\max}$ denotes the frame number of the maximum of the interpolating polynomial and $z_{step}$ is the step size of the piezo nanopositioner or motorized focus drive.

The lateral positions of the holes in calibration data set 1 or 2 are estimated by the least-squares fitting of a 2D Gaussian model [29] to the ROIs in the in-focus image of a z-stack. For calibration data set 3, the lateral positions are determined using all 200 in-focus images of the NanoGrid slide. Here, however, sample drift represents a more prominent effect due to the time required for the sequential acquisition of the 200 repeat images. Therefore, it needs to be corrected for in order to accurately determine the lateral hole positions. To correct for sample drift, the lateral positions of the holes are first estimated from each frame of the data set using the same fitting approach as for calibration data sets 1 and 2. Spatial transformations are then determined between the estimated hole positions for the first image and the corresponding estimated hole positions for the other images using the registration method of Section 2.5. The hole positions for each frame are then spatially transformed into the coordinate system of the first frame using the calculated transformation matrices. For each hole, the mean and standard deviation of its 200 spatially transformed coordinates are taken to be its lateral position and localization precision, respectively.

### 2.7 Measurement and adjustment of microscope stage and calibration sample horizontality

The tilt angle of the microscope stage is measured and adjusted as follows. The calibration sample is placed on the microscope stage for 30 minutes before imaging. This is to allow for temperature equilibration between the sample and the microscope to reduce sample drift. Applying the location estimation method of Section 2.6 to calibration data set 1 or a z-stack of calibration data set 2, the 3D position of each hole is estimated with high precision. A linear surface model is fitted to the estimated 3D positions using the least-squares criterion. The angle between the fitted plane z=ax+by+c and the horizontal plane z=0 is then calculated using their normal vectors. Furthermore, the difference between the highest and lowest estimated z positions is taken to be the maximum z position difference of the plane in the field of view. Using both the calculated angle and maximum z position difference, the horizontality of the stage is easily adjusted by inserting layers of paper or tape between the mounting frame and the stage at appropriate corners.

The above description, however, requires that the sample itself is flat. Otherwise, this method needs to be adjusted so that the stage is only horizontal if after a 180-degree rotation of the mounting frame with the sample, the measured angle has the same magnitude, but reversed sign.

Using the same approach, the horizontality of the calibration sample is measured and adjusted similarly by attaching layers of paper or tape on the bottom of the slide.

### 2.8 Comparison of data sets

The following analysis is carried out to measure the difference, in terms of the estimates of hole positions, between two calibration data sets acquired of the same NanoGrid sample under different conditions, including the use of different microscopes, different sample orientations, different illumination angles, and different detectors. In this analysis, calibration data set 3 is acquired using each setup and analyzed for comparison. The lateral position of each hole is estimated from each data set as described in Section 2.6 and the estimated positions are pair-matched to generate a spatial transformation matrix between the two data sets. For k=1,…,K, where *K* is the number of pairs of pair-matched holes, denote the *k*th pair-matched coordinates from the first data set (source data set) as $s_{k}\in {\mathbb{R}}^{2}$ and the *k*th pair-matched coordinates from the second data set (target data set) as $t_{k}\in {\mathbb{R}}^{2}$. Using these pairs of hole positions, the parameters of the affine transformation (i.e., the square invertible matrix $\hat{A}$ and the translation vector $\hat{d}$) are estimated as described in Section 2.5. The difference between the *k*th pair of hole positions is then defined as

$$e_{k}=t_{k}-[\hat{A}(s_{k})+\hat{d}],\;k=1,\;2,\;\ldots ,K.$$

**Fig. 1.**
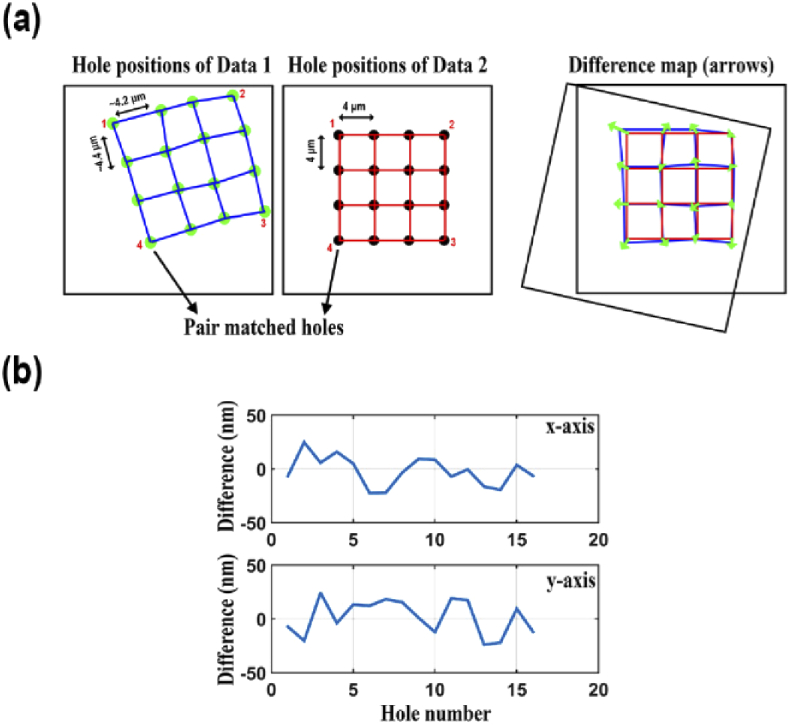
Illustration of difference analysis between pairs of hole positions. (a) Visualization of the difference map between two data sets. (b) Line plots of the differences along the x and y axes.

For visualization of the difference pattern, the differences between all *K* pairs of hole positions are represented with arrows as illustrated in the top panel of Fig. 1. The arrows in the difference map indicate the difference between each pair of hole positions, in terms of both direction and magnitude. The size of each arrow is chosen to be 200 times larger than the measured magnitude of the difference to allow for a straightforward visual inspection of the difference pattern. In addition, for a representation of the actual differences, the x and y components of the difference between each pair of hole positions are plotted as in the bottom panel of Fig. 1.

Finally, the root mean squared difference, which we call the “quality score” in this paper, is defined as

$$Q={\left(K^{-1}\sum_{k=1}^{K}{||e_{k}||}^{2}\right)}^{\frac{1}{2}}$$

where ||⋅|| denotes the Euclidean norm on ${\mathbb{R}}^{2}$. In some cases, a hole position estimate returned by the fitting of the 2D Gaussian model may be inaccurate. To make sure that these outlying estimates do not bias the score, we use a robust version of the quality score, obtained by excluding the two largest absolute positional differences from the calculation of *Q*. The quality score represents the overall difference between the two data sets in terms of the deviations between the positional estimates obtained for the NanoGrid holes. If there are significant deviations between corresponding hole positions from the two data sets, the quality score will be high. Conversely, if there are only small deviations between the corresponding hole positions, the quality score will be low.

### 2.9 Simulation of calibration data sets

**Fig. 2.**
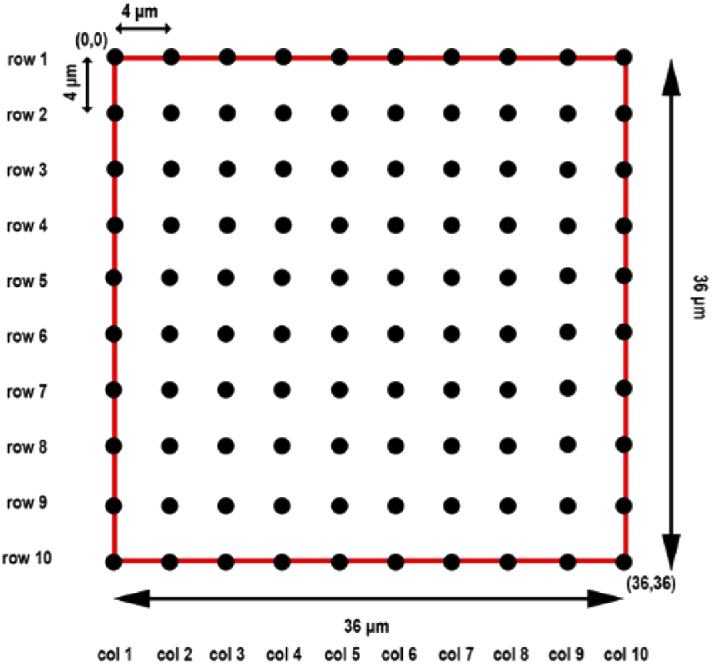
Schematic of the simulated 10 by 10 grid points.

The following simulation is carried out to generate simulated calibration data sets. We first generate 100 points that form a 10 by 10 grid with a uniform spacing of 4 µm between adjacent points (Fig. 2). Let $s_{k}\in {\mathbb{R}}^{2},\;k=1,\ldots ,100,$ denote the position of the *k*th grid point. The randomly distorted true position $t_{k}\in {\mathbb{R}}^{2}$ of the *k*th grid point is then modeled as

$$t_{k}=s_{k}+e_{k},k=1,\ldots ,100,$$

where $e_{k}=(e_{k,x},e_{k,y})$ with $e_{k,x}$ and $e_{k,y}$ the realizations of independent normal random variables with mean 0 and variance $\alpha^{2}$. The constant α indicates the overall geometric aberration level.

Subsequently, 200 repeat measurements of each distorted grid point position are generated from a normal distribution. Specifically, for k=1,…,100 and j=1,…,200, the *j*th repeat measurement ${\hat{t}}_{k,j}$ of the distorted position $t_{k}$ is given by

$${\hat{t}}_{k,j}=t_{k}+\epsilon_{k,j},$$

where $\epsilon_{k,j}=(\epsilon_{k,j,x},\;\epsilon_{k,j,y})$ represents the measurement error, with $\epsilon_{k,j,x}$ and $\epsilon_{k,j,y}$ the realizations of independent normal random variables with mean 0 and variance $\sigma^{2}$. The constant σ represents the localization uncertainty. The generation of a simulated calibration data set is carried out with a specific choice of the aberration level α and the localization uncertainty σ.

### 2.10 Accounting for experimental errors through repeat data acquisitions

Experimental errors intrinsic to the performance of repeat data acquisitions, such as errors associated with the focusing of a sample before each acquisition, places a practical limit on the best (i.e., the lowest) quality score that is attainable for a given microscopy setup. To determine this best quality score for a microscope, the following procedure is carried out. The horizontality of the microscope stage and the calibration sample is first determined and adjusted as described in Section 2.7. After acquiring 200 in-focus images of the NanoGrid slide (calibration data set 3), the precise lateral positions of the holes are estimated as described in Section 2.6. The sample is shifted along the x- or y-axis, and moved back to its original position. After refocusing, the lateral positions are again estimated after acquiring 200 more in-focus images of the NanoGrid slide. The quality score *Q* is then determined for the two data sets as described in Section 2.8. Importantly, the quality score *Q* determined for data from such repeat experiments is considered the lowest score $Q_{\min}$ that can be experimentally achieved for the given microscope configuration. It is used as a criterion in the determination of reference hole positions (Section 2.12).

Note that in the calculation of $Q_{\min}$, the determination of the affine transformation that registers the two sets of lateral hole positions includes the estimation of an angle of rotation, even though the sample is imaged with no change in its orientation after it is returned to its original position. The estimation of the rotation angle is justified, however, by the fact that the repositioning of the sample could potentially introduce a small rotation. Similarly, the estimation of the scale factor is justified by the fact that the refocusing of the sample after repositioning could potentially result in a slightly different magnification. To ensure the validity of the value obtained for $Q_{\min}$, one could always verify that the estimates for the rotation angle and the scale factor are reasonable for the given imaging setup.

### 2.11 Independence of estimated NanoGrid hole positions from the illumination angle

Two sets of 200 in-focus images of the NanoGrid slide (calibration data set 3) are acquired with two different angles of the illuminating light source, namely 0 and 45 degrees. The independence of the estimated hole positions from the illumination angle is then assessed by comparing the two data sets according to the procedure of Section 2.8. If the quality score *Q* is low enough, the estimated hole positions are considered independent from the illumination angle.

### 2.12 Determination of NanoGrid reference hole positions

Reference positions are accurately determined positions of the holes of a NanoGrid calibration sample that are used as a benchmark for comparison with other sets of positional estimates obtained for the same sample. To determine the reference positions, the horizontality of the microscope stage and the calibration sample is first determined and adjusted as described in Section 2.7. The precise lateral positions of the NanoGrid holes are then estimated as described in Section 2.6 after acquiring 200 in-focus images (calibration data set 3). The sample is rotated 180 degrees. Subsequently, the lateral positions are again estimated after acquiring 200 more in-focus images of the NanoGrid slide. The quality score *Q* is then determined for these two data sets using the procedure of Section 2.8. Whether this microscope is suitable for determining reference hole positions is evaluated using the quality score. If *Q* is low enough (i.e., close to $Q_{\min}$ or some value deemed acceptable for the intended use), the system is considered accurate enough for the determination of reference hole positions, and the hole positions estimated either before or after the 180-degree rotation can be used as reference positions for subsequent calibration analyses.

In addition to the rotation test, it is highly recommended that quality scores are determined for different positions of the NanoGrid slide in the field of view (occupied by the center 10 by 10 holes) to verify the absence of any significant field-dependent aberrations. Specifically, the slide should be positioned at locations that well sample the field of view. In each case, the quality score *Q* should be calculated with respect to the position at which the rotation test is performed, using the subset of holes that are within the bounds of the field of view for the translated slide position. The value of *Q* should satisfy a reasonable criterion in all cases in order to consider the system suitable for the determination of reference hole positions. A possible criterion for assessing a given score *Q* is its closeness to $Q_{\min}$ that is calculated with the same subset of holes.

Reference hole positions could alternatively be determined using higher-resolution imaging techniques such as atomic force microscopy and electron microscopy. The approach presented here, however, relies solely on an optical microscope and therefore has the advantage that no additional instrumentation is required.

### 2.13 Evaluation of optical components

#### 2.13.1 Axial chromatic aberration analysis for an objective lens

To analyze axial chromatic aberration for an objective lens, calibration data set 2 is acquired with three different color channels whose wavelengths approximately cover the range 400 nm to 700 nm. For example, the three channels commonly employed in fluorescence imaging experiments, namely the FITC, Cy3, and Cy5 channels used in microscope 2 (Section 2.2), are suitable choices. The horizontality of the calibration sample and the microscope stage is first determined and adjusted as described in Section 2.7. Using a motorized filter cube turret, an image of each of the three color channels is taken per z-step. A z-stack for each color channel, i.e., a total of three z-stacks, is thus acquired. To minimize mechanical drift after each z-step taken by the objective lens or each filter cube turret rotation, the acquisition software waits three seconds before acquiring the image. Given the three z-stacks, the axial positions of the NanoGrid holes are estimated as described in Section 2.6 for each channel. The axial hole positions for each channel are averaged, and the distance between the average axial hole positions for a pair of channels is taken to be the axial chromatic aberration between the two channels.

#### 2.13.2. Assessment of beam splitter/dichroic filter set performance

**Fig. 3.**
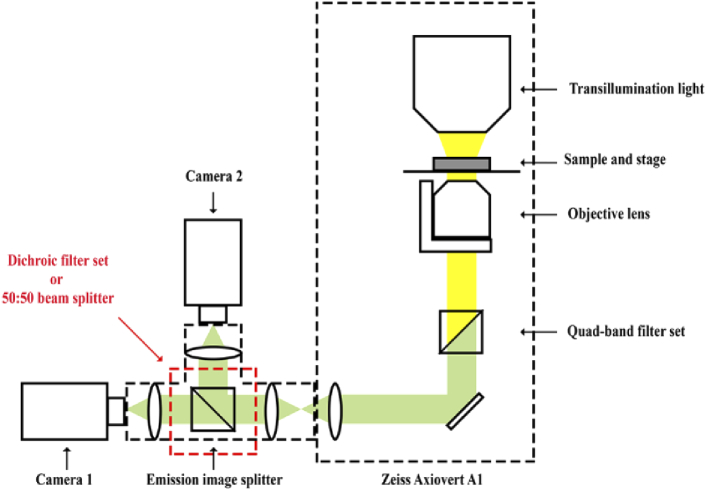
Illustration of a microscope setup for dual-color single-molecule imaging.

The performance of a dichroic filter/dichroic filter set or a beam splitter is assessed by comparing images acquired of its transmitted and reflected light paths. The simultaneous acquisition of the two data sets is carried out using two detectors, as illustrated in Fig. 3. Specifically, the performance of a dichroic filter/dichroic filter set or beam splitter is analyzed as follows. The horizontality of the calibration sample and the microscope stage is first determined and adjusted as described in Section 2.7. After mounting the filter set or beam splitter in the emission image splitter, the precise lateral positions of the NanoGrid holes are independently estimated, as described in Section 2.6, from 200 in-focus images of the calibration slide (calibration data set 3) acquired by camera 1 (in the path of the transmitted light) and camera 2 (in the path of the reflected light). The quality score *Q* is then computed for the two data sets using the procedure of Section 2.8. The performance of the dichroic filter/dichroic filter set or beam splitter is then assessed based on the quality score. A low enough value for *Q* indicates little difference between the images produced by the transmitted and reflected light paths, and the dichroic filter/dichroic filter set or beam splitter is considered to be of high quality. Note that it is important for this analysis that in-focus images are simultaneously acquired by the two cameras. Even though the cameras have been aligned so that they share a focal plane (Section 2.2), it is advisable to verify that the in-focus positions for the two cameras, determined by applying the Brenner gradient method separately to z-stacks acquired by the cameras, are in fact the same. This verification should be done before moving the objective lens to the common in-focus position and carrying out the simultaneous acquisition of the 200 images by the two cameras.

### 2.14 Software

In the acquisition of data sets for the demonstration of our calibration method, components such as the camera, the piezo nanopositioner, and the filter cube turret were controlled and synchronized using custom software written in the C programming language. The acquired data were processed and analyzed using custom programs written in MATLAB (The Mathworks, Inc) as well as the image analysis software Lumio (Astero Technologies LLC).

## 3. Results

In this section, we demonstrate our calibration method by applying its constituent tools and protocols to specific microscope setups and optical components. Details pertaining to the calibration sample, the instrumentation, the data sets, and the tools and protocols are all as provided in Section 2.

### 3.1 Acquisition of z-stack images of a calibration sample

**Fig. 4.**
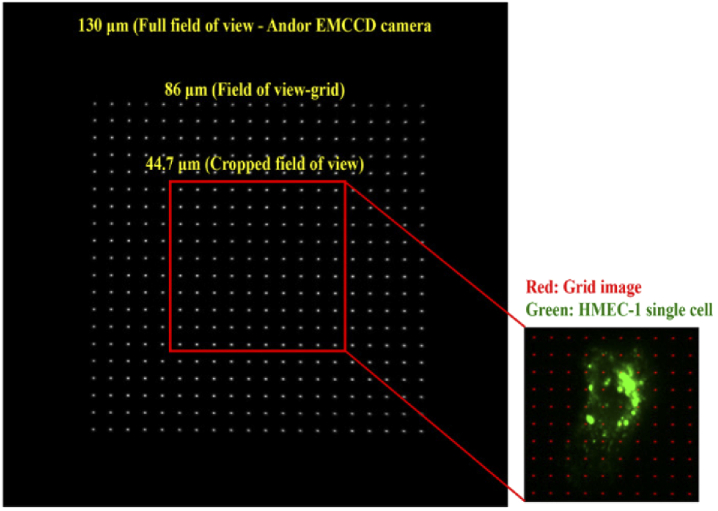
Full camera field of view capturing an entire NanoGrid slide. The image shown is the in-focus image from a 50-image z-stack acquired using microscope 1. The red box delineates the cropped field of view that is used for analysis.

The process of acquiring a z-stack of a NanoGrid sample (Section 2.1) is essential for obtaining the three types of calibration data described in Section 2.3. Microscope 1 (Section 2.2) was used to acquire, in steps of 50 nm, a z-stack of a NanoGrid slide consisting of 50 images (calibration data set 1, Section 2.3). In this data set, the center 10 by 10 NanoGrid holes to be used for analysis occupy a field of view of 44.7 µm × 44.7 µm. Since human microvascular endothelial (HMEC-1) cells cultured on coverslips are typically not larger than 45 µm, this cropped field of view is large enough to capture an entire HMEC-1 cell, as shown in Fig. 4.

### 3.2 Determination of the in-focus image of the sample

**Fig. 5.**
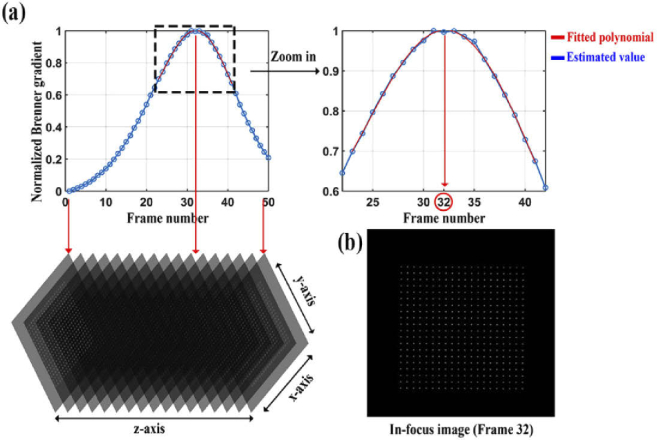
Determination of the in-focus image of the sample. (a) A polynomial fit of the normalized Brenner gradient as a function of the z-stack frame number. (b) In-focus image of the sample.

Determining an in-focus position is important for precisely estimating the lateral positions of the NanoGrid holes. This is because the closer the image is to focus, the higher the localization precision [30]. The determination of the in-focus image of the z-stack from Section 3.1 was carried out using the method based on the Brenner gradient (Section 2.4). Figure 5(a) shows the plot of the normalized Brenner gradient versus the z-stack frame number, smoothed by a fourth-order polynomial fitted around the maximum of the plot. Since the frame number closest to the maximum of the interpolating polynomial is 32, the 32nd image of the z-stack, shown in Fig. 5(b), is taken to be the in-focus image of the sample.

### 3.3 Estimation of lateral hole positions

**Fig. 6.**
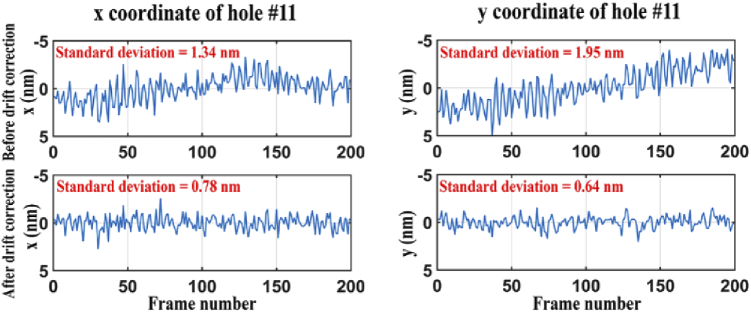
Plots showing the estimated x and y positions of an arbitrarily chosen hole over 200 frames, before and after drift correction.

Our calibration method entails analyses that rely on the precise estimation of the positions of NanoGrid holes. In order to estimate the lateral position of the holes with high precision, we need to acquire a significant number of photons. Therefore, using microscope 1, 200 images of the NanoGrid slide (calibration data set 3) were acquired at the in-focus position determined in Section 3.2 with high image brightness. Using the procedure specified in Section 2.6, we then estimated the lateral positions of the NanoGrid holes. Due to the time it takes to sequentially acquire 200 images, correction for sample drift represents an important part of the position estimation procedure. Figure 6 shows, for a randomly selected hole, the x and y positions estimated from the 200 frames by the fitting of a 2D Gaussian model, before and after drift correction. The standard deviations of the x and y positions before and after drift correction are [1.34 nm, 1.95 nm] and [0.78 nm, 0.64 nm], respectively. This demonstrates that the lateral hole position can be estimated more precisely, by about 41.8% and 67.2% for x and y in this particular case, after drift correction.

**Fig. 7.**
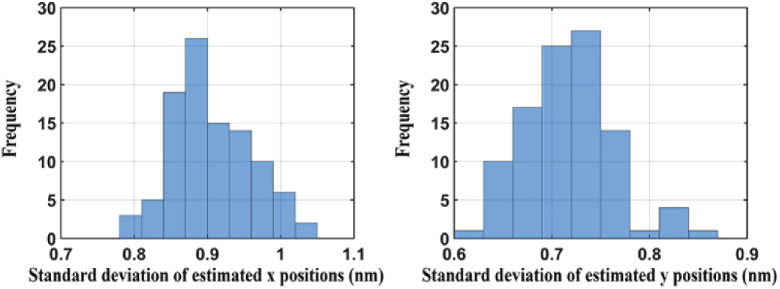
Histograms of the standard deviations of the x and y positional estimates for all 100 holes analyzed.

Figure 7 shows histograms of the standard deviations of the x and y estimates for all 100 holes. We can see that, for this data set, the localization precision for each hole is less than 1.05 nm and 0.9 nm in the x and y directions, respectively. In addition, the differences between the minimum and maximum standard deviations of the x and y estimates are 0.25 nm and 0.24 nm, respectively, showing that the localization precision is relatively uniform throughout the cropped field of view.

### 3.4 Estimation of axial hole positions

**Fig. 8.**
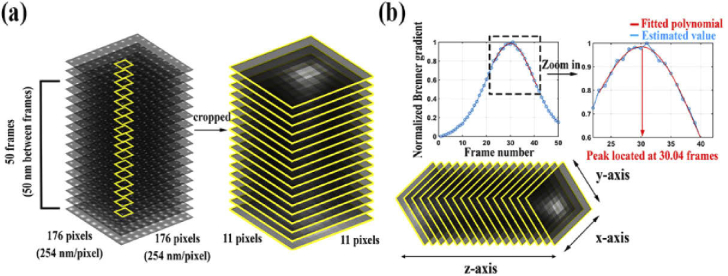
(a) An example of an ROI z-stack for a hole. (b) Estimation of the axial position of a hole.

As described in Section 2.7 and Section 2.13.1, from a calibration data set consisting of one or more z-stacks (i.e., calibration data set 1 or 2), the axial positions of the NanoGrid holes are estimated and used to determine the tilt angle of the stage and sample and the chromatic aberration of an objective lens. To demonstrate the task of estimating axial hole positions, we followed the procedure of Section 2.6 and began by extracting as many ROI z-stacks as there are holes from the z-stack data set of Section 3.1. This extraction of ROI z-stacks is illustrated in Fig. 8(a). For each hole, the axial position was estimated, as explained in Section 2.6, by applying the Brenner gradient-based method for in-focus frame determination to the hole’s ROI z-stack. As shown in Fig. 8(b) for an arbitrarily chosen hole, since the maximum of the interpolating polynomial from the Brenner gradient-based method occurs at 30.04 frames, the hole’s z position is estimated to be 1452 nm, the product of (30.04–1) and the piezo nanopositioner step size of 50 nm.

**Fig. 9.**
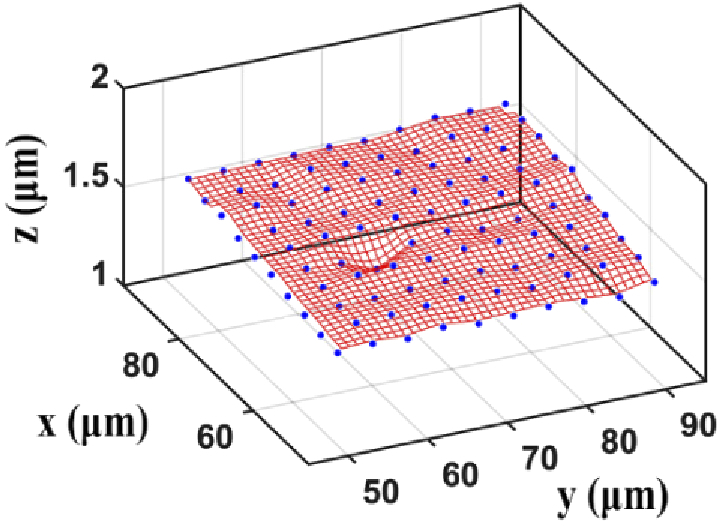
A wireframe 3D mesh plot determined by the 3D positions of 100 NanoGrid holes. The blue dots represent the estimated positions of the NanoGrid holes. The wireframe mesh facilitates the visualization of the differences between the z positions of the holes. It is obtained by linearly interpolating the hole positions using a finer grid.

The 3D position of each hole was then given by its estimated z position and its lateral position. As specified in Section 2.6, the lateral position was estimated by fitting a Gaussian model to the hole’s ROI from the in-focus frame of the z-stack. A wireframe 3D mesh plot showing the 3D positions of all 100 holes is presented in Fig. 9.

### 3.5 Horizontality adjustment of microscope stage and calibration sample

**Fig. 10.**
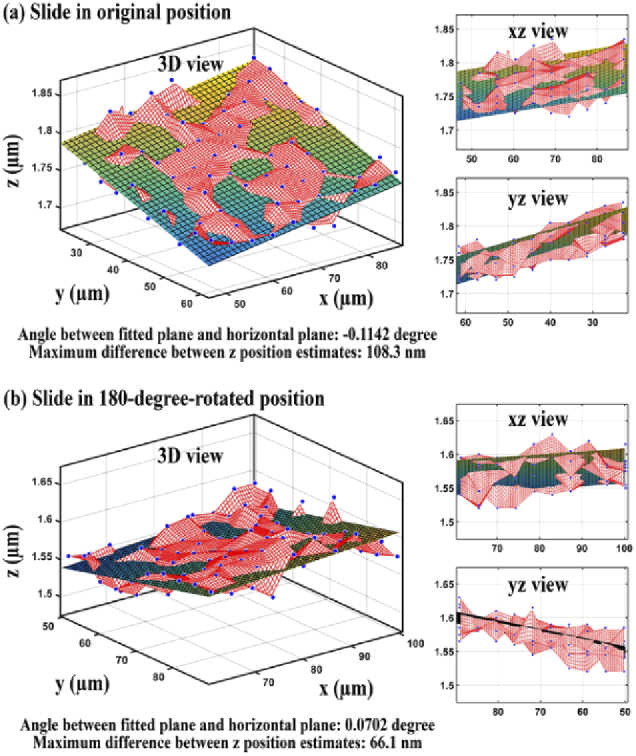
Estimated 3D positions of 100 holes (blue dots) and the fitted plane when (a) the NanoGrid slide is in its original position and (b) the NanoGrid slide is in its 180-degree-rotated position.

Wide-field microscopy typically requires the microscope stage to be perpendicular to the optical axis. Therefore, it is necessary to determine the horizontality of the microscope stage and make any needed adjustments. The horizontality of the stage on microscope 1 was measured as described in Section 2.7. Figure 10(a) shows, from different viewpoints, the 3D positions of the NanoGrid holes (blue dots) and the fitted plane. The angle between the fitted plane and the horizontal plane and the maximum z difference of the plane in the field of view were determined to be −0.1142° and 108.3 nm, respectively. As we could not assume the NanoGrid slide to be flat, we performed the same analysis after a 180-degree rotation of the mounting frame with the sample. The results are shown in Fig. 10(b), and in this case, the angle and the maximum z difference were found to be 0.0702° and 66.1 nm, respectively.

**Fig. 11.**
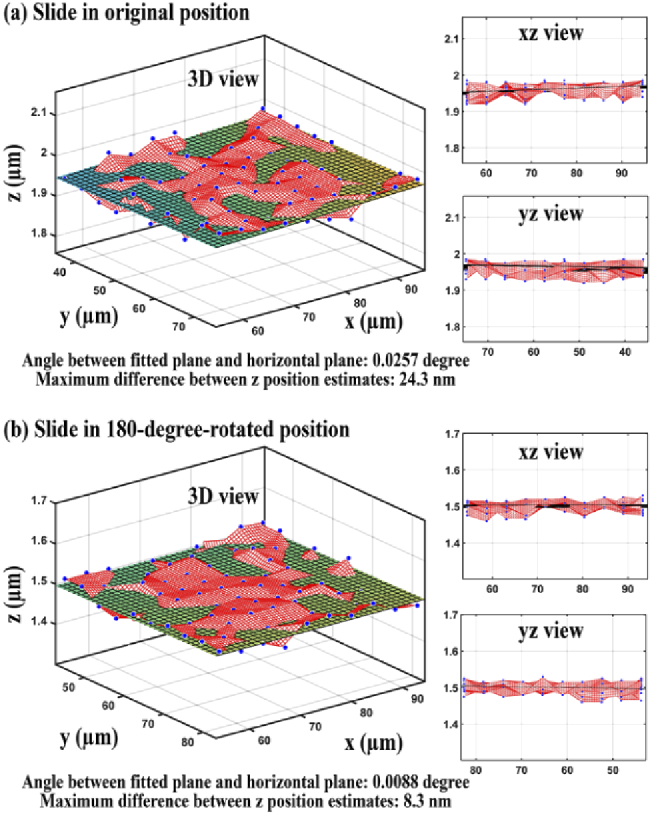
Estimated 3D positions of 100 holes (blue dots) and the fitted plane when (a) the NanoGrid slide is in its original position and (b) the NanoGrid slide is in its 180-degree-rotated position.

The above results show that while the sign of the tilt angle was reversed after the 180-degree rotation of the mounting frame, the magnitude of the angle had decreased. This indicated that the stage or the sample or both were not horizontal. Therefore, we adjusted the horizontality of the stage and sample as described in Section 2.7 and repeated the same analysis. Figure 11(a) shows the results for the data acquired in the original orientation of the sample after adjustment. The angle between the horizontal plane and the plane fitted to the 3D positions of the NanoGrid holes was found to be 0.0257° and the maximum z difference of the plane in the field of view was determined to be 24.3 nm. Figure 11(b) shows that for the data acquired in the 180-degree-rotated orientation of the NanoGrid slide, the angle and the maximum z difference were found to be 0.0088° and 8.3 nm, respectively. These results show that, after adjustment, the slopes before and after rotating the sample are similar and close to zero. In other words, both the microscope stage and the calibration sample can now be considered to be horizontal.

### 3.6 Simulation study investigating the dependence of the quality score *Q* on the extent of geometric aberration and localization uncertainty

In this section, we investigate the dependence of the quality score *Q* (see Section 2.9) on the uncertainty in repeat measurements of a NanoGrid hole position (i.e., the localization precision) and the overall level of geometric aberration in the microscope system.

We carried out two simulation studies. For the first simulation study (simulation study 1), two calibration data sets were generated as described in Section 2.9 for each scenario considered. The scenarios differ in terms of the position measurement uncertainty σ and the level of geometric aberration α, the values of which are given in Table 1. For each scenario, the first data set was simulated with no geometric aberrations (i.e., α=0 for the first data set). Geometric aberrations were simulated only in the second data set. Both data sets were generated with the same position measurement uncertainty.

**Table 1.** Parameter values used for simulation study 1

Simulation parameter	Range	Increment
Uncertainty of position measurements σ (nm)	0–20	2
Level of geometric aberration α (nm)	0–9	1

**Fig. 12.**
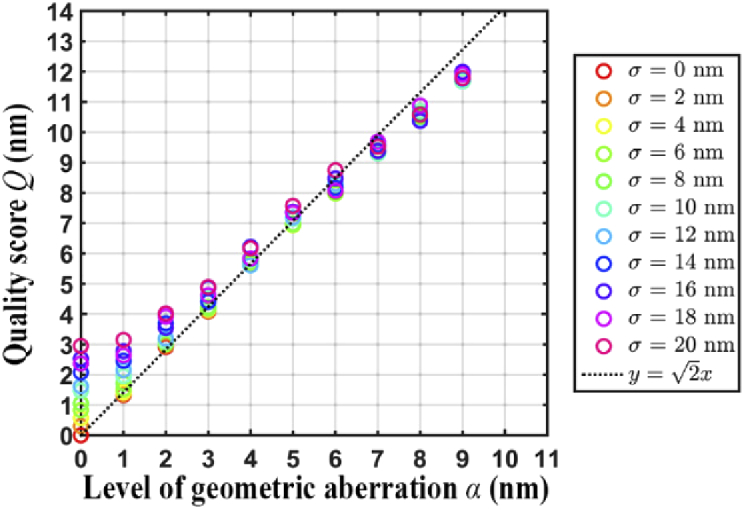
Results of simulation study 1, illustrating the dependence of the quality score *Q* on the level of geometric aberration α and localization uncertainty σ.

Figure 12 summarizes the quality score *Q* for all scenarios considered. The x-axis of the plot represents the overall geometric aberration level α and the y-axis represents the calculated quality score *Q*. Different colors are used to denote different values of the localization uncertainty σ. We see from the plot that the quality score *Q* increases with the overall geometric aberration level α. This is as expected, since larger values of α result in larger deviations between the hole positional estimates obtained for the two data sets. The plot also indicates that for a given aberration level α, *Q* increases with the localization uncertainty σ. In particular, when α is small, comparatively large values of the localization uncertainty can increase the value of *Q* significantly. Also shown in the plot is the line $y=\sqrt{2}x$. This line is intended as a reference for comparison, since for the data model assumed here, the quality score *Q* is effectively an estimate of $\sqrt{2}\alpha$ when there is no localization uncertainty (i.e., when σ=0) and when one of the data sets is without geometric aberration. Specifically, under these conditions, *Q* is an estimate of the quantity $\sqrt{Var(X)+Var(Y)}=\sqrt{\alpha^{2}+\alpha^{2}}=\sqrt{2}\alpha$, where *X* and *Y* are Gaussian random variables with variance $\alpha^{2}$. Indeed, for the data sets analyzed here, we can see that, especially for smaller values of α, *Q* is relatively close to $\sqrt{2}\alpha$ when σ is small.

The second simulation study (simulation study 2) was carried out the same way as the first study, but with a different range of localization uncertainties. For the second study, the localization uncertainty σ was varied over a more realistic, small-valued range based on what we have observed with our experimental data (see Table 2). For the data sets analyzed in this simulation study, we can see that *Q* is very close to the $\sqrt{2}\alpha$ benchmark for all values of α considered.

**Table 2.** Parameter values used for simulation study 2

Simulation parameter	Range	Increment
Uncertainty of position measurements σ (nm)	0–1.4	0.2
Level of geometric aberration α (nm)	0–9	1

**Fig. 13.**
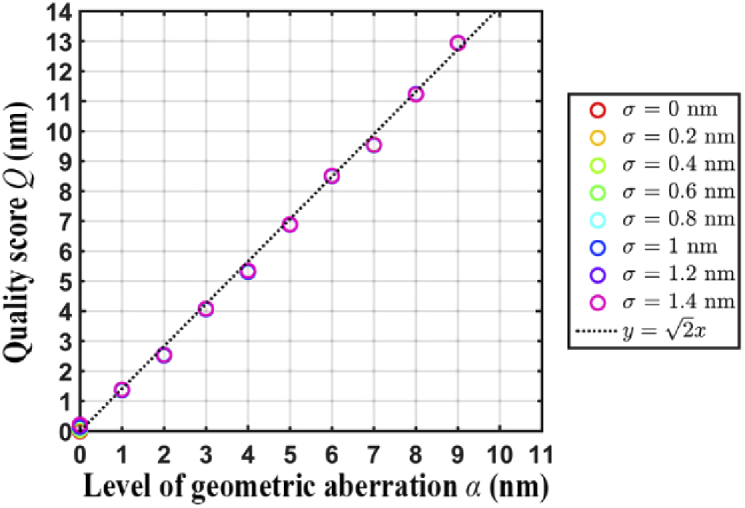
Results of simulation study 2, which considers significantly smaller but more realistic localization uncertainties σ than simulation study 1.

Similar to Fig. 12, Fig. 13 demonstrates, for simulation study 2, that as the overall geometric aberration level α increases, the quality score *Q* also increases. However, Fig. 13 shows that when the localization uncertainty σ is small, it has negligible effect on the value of *Q*.

The results of our simulation studies indicate that in order to obtain quality scores that accurately reflect the difference in the level of geometric aberration between two data sets, it is important that the NanoGrid hole positions are estimated with high precision (i.e., low localization uncertainty) in both data sets.

### 3.7 Accounting for experimental errors with baseline quality score

**Fig. 14.**
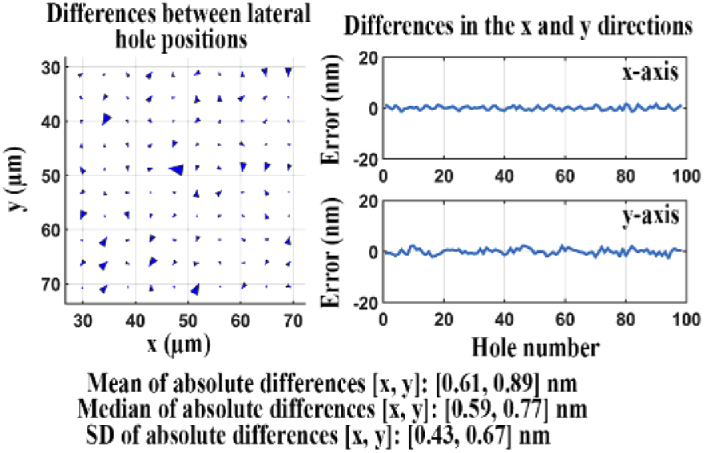
Difference analysis for data sets acquired before and after repositioning of the NanoGrid slide.

To demonstrate the calculation of the baseline quality score $Q_{\min}$, we carried out the analysis procedure described in Section 2.10. First, we acquired 200 in-focus images of the NanoGrid slide (i.e., calibration data set 3) using microscope 1. We then moved the sample about 40 µm to the right and then back to its original position. Following this repositioning, we refocused the sample and again acquired 200 in-focus images using the same acquisition settings as before. We then determined the baseline quality score $Q_{\min}$ according to the procedure described in Section 2.8. Note that the effects of geometric aberration are solely related to the positional coordinates of the NanoGrid holes. For both data sets, since the same sample was imaged in the same position, through $Q_{\min}$ we can account for experimental errors that are intrinsic to the acquisition of data, unmingled with the effects of geometric aberration. The left panel of Fig. 14 shows a plot of arrows indicating the differences between all 100 pairs of corresponding hole positions estimated from the two data sets. The right panel of Fig. 14 shows plots of the differences in the x and y directions for each pair of corresponding positions. Based on these positional differences, the quality score $Q_{\min}$ between the repeat acquisitions was determined to be 1.34 nm. This value is considered the lowest score (i.e., the best score) that can be achieved with the microscope configuration used.

### 3.8 Independence of NanoGrid hole position measurements from the illumination angle

As the NanoGrid slide was illuminated with a LED light source, we wanted to make sure that specifics of the illumination did not impact the results. We therefore imaged the same slide using two different illumination angles of the light source and compared the hole positions estimated from the two data sets.

**Fig. 15.**
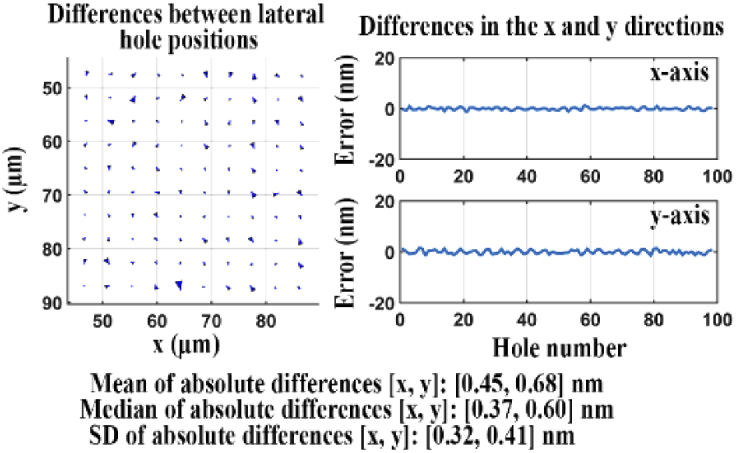
Difference analysis for data sets acquired with 0-degree and 45-degree illumination light.

The procedure followed is as described in Section 2.11. In-focus images of the sample were first acquired using microscope 1 with a 0-degree illumination angle (i.e., the illumination light rays traveled parallel to the optical axis and perpendicular to the microscope stage). This was followed by another acquisition of in-focus images of the sample using the same microscope, but with a 45-degree illumination angle. The two data sets were then analyzed as described in Section 2.8. The left panel of Fig. 15 shows a plot of arrows representing differences between the corresponding hole positions estimated from the two data sets. The right panel of the figure shows line plots of the differences in the x and y directions for each pair of corresponding positions.

The quality score *Q* for the two data sets is 0.96 nm, which is lower than $Q_{\min}$ measured in Section 3.7. This can be explained by the fact that the second data set here was acquired without first repositioning the sample or the stage. The baseline score $Q_{\min}$, however, accounts for the experimental error that is necessarily introduced when the sample is moved and refocused between acquisitions, and therefore reflects a poorer but more realistic benchmark for what can typically be expected in practice. The result indicates that the overall positional deviation between the two data sets is less than 1 nm, and therefore that the angle of illumination does not impact the measurement of the NanoGrid hole positions.

### 3.9 Determination of reference positions of NanoGrid holes

We propose the use of a NanoGrid slide as a calibration standard to evaluate the performance of a microscope system or optical components such as the objective lens and the dichroic filter. The approach is to determine whether the microscope or optical component in question causes geometric aberrations in the light path that are reflected in deviations of hole position estimates from their true positions. As we can expect manufacturing errors in the positions of the NanoGrid holes, we cannot take the holes’ nominal positions to be their true positions. Instead, it is necessary for us to determine accurate locations of the holes ourselves, and use them as a reference for comparison. Specifically, we need to be able to very precisely estimate the positions of the holes using a “high-quality” microscope. A high-quality microscope is one that does not introduce significant distortions in the measured hole locations. Therefore, the hole position estimates obtained using such a microscope can be used as reference hole positions for subsequent calibration analyses.

The approach used to establish whether a microscope is suitable for the determination of reference hole positions is as follows. Suppose the calibration sample is imaged before and after a rotation or translation. Any specific pattern associated with the sample should move with the sample, but a geometric aberration pattern should not change with the position or orientation of the sample. This is because geometric aberrations are caused by defects or misalignments in the optics, and not due to variations in the sample. Provided that we use a high-quality microscope, the imaged pattern after the rotation or translation should coincide with the pattern before the rotation or translation. On the other hand, when we use a microscope that distorts the measured locations of the holes, the imaged patterns before and after the rotation or translation will not match well.

Using this concept, the reference positions of the NanoGrid holes were determined as described in Section 2.12. Specifically, we acquired 200 in-focus images of the NanoGrid slide (calibration data set 3) using microscope 1, before and after rotation of the sample by 180 degrees. We then compared the two data sets using the procedure of Section 2.8. Note that here we omitted the tests described in Section 2.12 that involve the translation of the slide to sample different locations of the field of view.

**Fig. 16.**
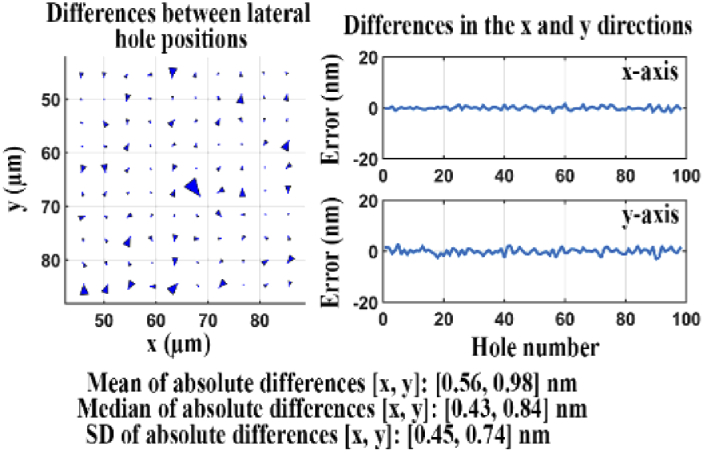
Difference analysis for data sets acquired before and after slide rotation using microscope 1.

The left panel of Fig. 16 shows a plot of arrows indicating the differences between the corresponding hole positions estimated from the two data sets, while the right panel shows plots of the x and y components of the differences. The quality score *Q* for the two data sets is 1.42 nm, which is very close to the baseline score $Q_{\min}$ measured in Section 3.7. To be precise, it is just 1.06 times larger than $Q_{\min}$. We therefore conclude that microscope 1 is suitable for determining reference hole locations, and we take the estimated positions before rotation of the sample as reference hole positions for evaluations of a microscope system or optical component that utilize the same NanoGrid slide.

### 3.10 Detection of geometric aberration in the light path

Many microscope imaging systems make use of an objective thread adapter that allows an objective lens to be used on a microscope with a mounting thread that is not of the same pitch or diameter as the objective lens. Such adapters are also often used to increase the axial position of the objective lens. If an inappropriate adapter, such as one that is damaged or one whose thread is incompatible with the thread of the objective lens, is used in a setup, geometric aberrations could be introduced.

To illustrate the analysis of a microscope configuration that exhibits geometric aberration (i.e., a “poor quality” microscope), we installed a damaged thread adapter for use with the objective lens on microscope 1. The evaluation of the microscope system was then carried out as follows, using the reference NanoGrid hole positions determined in Section 3.9. The horizontality of the sample and microscope stage was first determined and adjusted using the procedure of Section 2.7. We then acquired 200 in-focus images of the NanoGrid slide (calibration data set 3) using this poor quality microscope, and precisely estimated the lateral positions of the NanoGrid holes according to Section 2.6. Subsequently, the analysis of the difference between the estimated hole positions and the reference positions was carried out as described in Section 2.8.

**Fig. 17.**
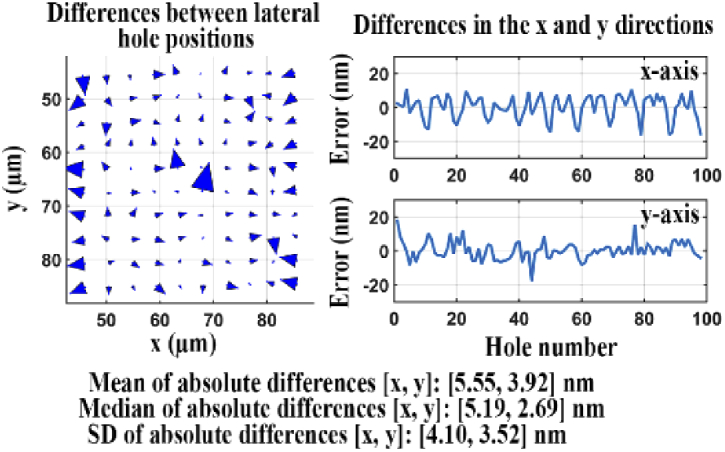
Difference analysis for two data sets, one acquired using a reference microscope, the other acquired using an inadequate microscope.

An arrow plot showing the differences between the estimated hole positions and their corresponding reference positions is provided in the left panel of Fig. 17. The right panel of the figure shows line plots of the x and y components of the differences. The quality score *Q* for the two sets of lateral positions is a large 8.67 nm, indicating that the microscope configuration indeed produces significant geometric aberrations.

**Fig. 18.**
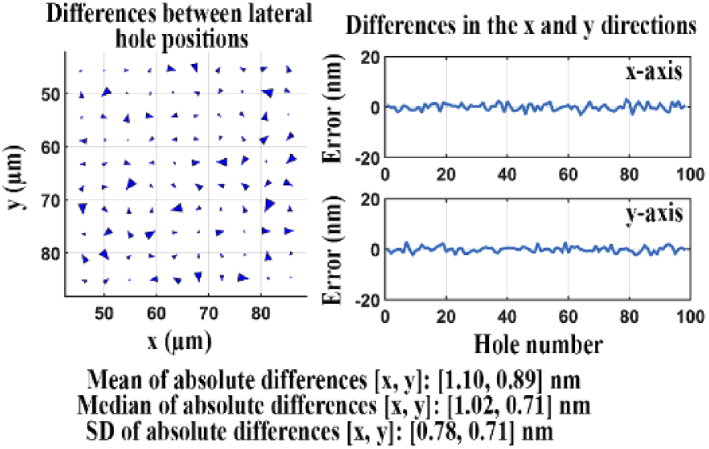
Difference analysis for two data sets, one acquired using a reference microscope, the other acquired using a comparable microscope.

We carried out the same analysis to evaluate a different microscope configuration (microscope 2, Section 2.2) and verified that the microscope introduces only a very minor distortion of the hole positions. The much improved results are shown in Fig. 18, and the quality score *Q* for the two sets of lateral positions is a significantly smaller 1.76 nm. The small quality score confirms that this microscope configuration introduces only minor geometric aberrations.

### 3.11 Evaluation of optical components

#### 3.11.1 Evaluation of axial chromatic aberration of an objective lens

**Fig. 19.**
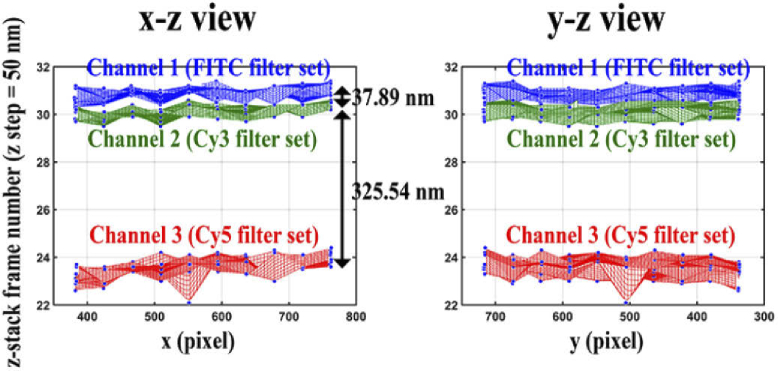
Analysis of the axial chromatic aberration introduced by an objective lens.

Axial chromatic aberration occurs due to light of different wavelengths refracting differently when propagating through the optical path of a microscope. Even expensive objective lenses may not be fully corrected for wide spectral ranges. It is therefore necessary to analyze an objective lens for axial chromatic aberration. We evaluated the objective lens of microscope 2 using filter sets for three different wavelength ranges, following the procedure detailed in Section 2.13.1. The axial and lateral positions of the NanoGrid holes were estimated as described in Section 2.6 for each of the three different wavelength ranges considered. Figure 19 shows the wireframe mesh plots of the 3D hole positions estimated for the three color channels. The red, green, and blue colors correspond to the results obtained with the FITC, Cy3, and Cy5 filter sets, respectively. The distance between the averages of the axial hole positions for two channels represents the axial chromatic aberration between those channels. As we can see in Fig. 19, the axial shift between channel 1 (FITC) and channel 2 (Cy3) is 37.89 nm, and the axial shift between channel 2 and channel 3 (Cy5) is 325.54 nm. These results show that the objective lens is not fully corrected when working across a wide spectral range. The aberration is especially severe between the near-infrared range and the blue and green ranges. The axial shift of 37.89 nm between channel 1 and channel 2 cannot be ignored in studies requiring short distance measurements between the two different color probes. The significantly larger axial shift of 325.54 nm between channel 2 and channel 3 could lead to even more serious consequences in any multicolor experimental application. It is therefore important to measure such axial shifts and to take them into consideration when analyzing data acquired in a multicolor experiment.

#### 3.11.2 Performance assessment of a dichroic filter set

Cubes pre-mounted with a filter set are available that can be easily integrated into an emission image splitter unit to separate signals of interest by wavelength. A filter set placed in the image splitter typically consists of a dichroic filter and two emission filters.

Unlike conventional microscopy, single-molecule imaging systems are very sensitive to optical wavefront distortions that are potentially caused by a non-flat dichroic filter. The flatness of the dichroic filter has no effect on the light transmitted through the filter, but an insufficiently flat dichroic filter often distorts the wavefront of the light it reflects. Dichroic filters with improved flatness may minimize geometric aberrations in the reflected beam that are obtained with standard dichroic filters. The geometric aberrations caused by a dichroic filter can be detected using the difference between the image transmitted through the filter and the image reflected by the filter. We describe here an application of the approach to dichroic filters of two different types of flatness, standard and improved.

##### 3.11.2.1 Standard flatness dichroic filter set

The concept used to determine the quality of a dichroic filter is as follows. Suppose that the calibration sample is imaged with camera 1 in the path of the transmitted emission beam and camera 2 in the path of the reflected emission beam, as shown in Fig. 3. Provided that the dichroic filter is “perfect”, the imaged pattern from camera 2 should match the imaged pattern from camera 1. On the other hand, if an imperfect dichroic filter is used, the imaged pattern from camera 2 will not coincide with the imaged pattern from camera 1 due to distortions introduced by the filter.

Using this concept, the evaluation of the standard flatness dichroic filter set was carried out using microscope 3 as described in Section 2.13.2. After mounting the standard flatness dichroic filter set, 200 in-focus images of the NanoGrid slide (calibration data set 3) were separately but simultaneously acquired by camera 1 and camera 2. Two sets of lateral positions were estimated from the two data sets, and the difference between them was analyzed according to Section 2.8. Note that by applying the Brenner gradient method of Section 2.4 to z-stacks acquired by the two cameras, the cameras’ in-focus positions were found to differ by just 0.18 frames, or 9 nm given the z-stack step size of 50 nm. The simultaneous acquisition of the 200 images by the two cameras was therefore carried out at a common in-focus position.

**Fig. 20.**
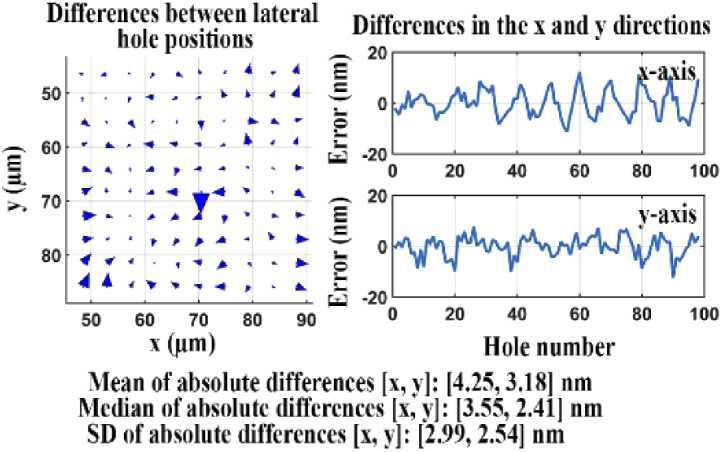
Difference analysis for data sets acquired of the transmitted and reflected light paths of a standard flatness dichroic filter.

Figure 20 illustrates the differences between the lateral hole positions obtained from the data acquired by the two cameras. The quality score *Q* for the two sets of lateral positions is 6.58 nm, which in this case indicates the overall aberration level in the reflected light path. The relatively large value of *Q* indicates that this standard flatness filter set is of insufficient quality for nanometer-scale dual color imaging.

##### 3.11.2.2 Improved flatness dichroic filter set

**Fig. 21.**
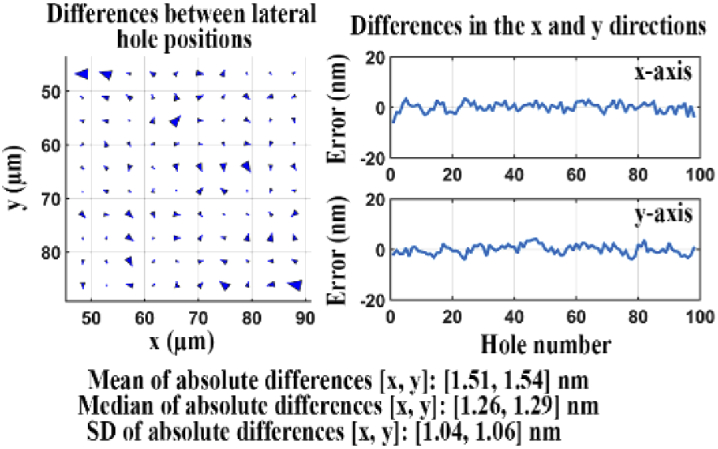
Difference analysis for data sets acquired of the transmitted and reflected light paths of an improved flatness dichroic filter.

We carried out the same analysis again using microscope 3, but for a dichroic filter with improved flatness. (In this case, the in-focus positions of the two cameras were also verified to differ by just 9 nm.) The results are shown in Fig. 21. In this case, the quality score *Q* for the two sets of estimated lateral hole positions is 2.62 nm. This quality score is significantly smaller than that for the standard flatness dichroic filter, indicating that the improved flatness dichroic filter introduces some distortion in the reflected light path, but to a lesser extent than the standard flatness dichroic filter. Therefore, the improved flatness dichroic filter set should be preferred for experiments that require, for example, nanometer-scale distance measurements between two probes of different colors.

## 4. Conclusion

The advent of single-molecule microscopy has enabled researchers to estimate the position of single molecules at the nanoscale, allowing the tracking of the dynamics of single molecules and the visualization of fine details of subcellular structures. At such high resolutions, calibration of the microscope system plays an important role in guaranteeing the appropriate level of accuracy for the results obtained. Calibration should be one of the essential steps in the design and implementation of single-molecule imaging experiments, and it needs to be carried out with great care. We therefore developed a calibration method that is optimized for single-molecule microscopy, and in particular for multicolor applications.

Our calibration method is based on detecting geometric distortions in a microscope’s light path by imaging a NanoGrid slide, which comprises an array of regularly spaced sub-wavelength-sized holes. The quality of a microscope is assessed by estimating the hole locations and evaluating the deviation of the locations from reference hole positions that have been obtained using a high-quality microscope. We showed here that when enough light is collected, hole positions can be determined with a sub-nanometer standard deviation, thereby allowing calibration at the nanometer scale. By applying the method to microscopes of good and poor quality, we demonstrated that our approach can indeed be used to evaluate microscope performance.

The proposed calibration method also includes approaches for evaluating the quality of optical components. The dichroic filter is a critical component in multicolor single-molecule microscopy, and any distortions it introduces can be highly problematic for multicolor single-molecule experiments. The use of a dichroic filter of insufficient quality can lead to significant distortions in the reflected light path. In our approach, the quality of a dichroic filter is evaluated by comparing the positions of NanoGrid holes estimated from data acquired of the filter’s transmitted and reflected light paths. We applied our method to dichroic filters of differing flatness, and showed that it can in fact discern their performance.

In addition, we presented an approach for assessing the axial chromatic aberration introduced by an objective lens. Axial chromatic aberration refers to focus shift as a function of the wavelength of the collected light. In multicolor imaging experiments, even slight focus shifts between colors can result in misinterpretation of the acquired data. This is especially the case in the single-molecule context. Since most objective lenses are designed to correct for chromatic aberration (i.e., they are designed to be apochromatic), the importance of this evaluation is often overlooked. Even apochromatic objective lenses do not necessarily provide the level of correction needed over a wide spectral range, and our demonstration shows that such lenses can in fact introduce significant chromatic aberration.

The proposed calibration method is relatively easy to implement, and therefore suitable for routine assessments of single-molecule imaging setups in a laboratory. By imaging and localizing the sub-wavelength-sized holes of a NanoGrid slide or similar calibration standard, the user can easily follow the protocol to obtain a quality score that quantifies the level of geometric aberration that is produced by the given imaging configuration. At the core of the method is a general procedure that can be applied not only to the microscope as a whole, but also to a specific optical element as we have demonstrated with dichroic beam splitters.
